# Harmonics Generation in the Laser-Induced Plasmas of Metal and Semiconductor Carbide Nanoparticles

**DOI:** 10.3390/nano12234228

**Published:** 2022-11-28

**Authors:** Vyacheslav V. Kim, Srinivasa Rao Konda, Weili Yu, Wei Li, Rashid A. Ganeev

**Affiliations:** 1 The GPL Photonics Laboratory, State Key Laboratory of Applied Optics, Changchun Institute of Optics, Fine Mechanics and Physics, Chinese Academy of Sciences, Changchun 130033, China; 2 Laboratory of Nonlinear Optics, Institute of Astronomy, University of Latvia, Jelgavas Iela 3, LV-1004 Riga, Latvia; 3 Tashkent Institute of Irrigation and Agricultural Mechanization Engineers, National Research University, Kori Niyozov Street 39, Tashkent 100000, Uzbekistan; 4 Department of Physics, Voronezh State University, 394006 Voronezh, Russia

**Keywords:** B_4_C, Cr_3_C_2_, SiC, high-order harmonics generation, laser-induced plasma, chirped pulses

## Abstract

Carbon-containing plasma is an attractive medium for generation of harmonics of laser pulses in the extreme ultraviolet range. We ablate two metal carbide (B_4_C and Cr_3_C_2_) nanoparticles and silicon carbide (SiC) nanoparticles and generate harmonics after propagation of 35 fs pulses through the laser-induced plasmas. We analyze the spectra, spectral shifts, and splitting of harmonics from nanoparticles-contained plasmas, which demonstrate the chirp-related harmonic cut-off scaling. In addition, we present the simplified two-color pump model calculations of HHG based on the strong field approximation.

## 1. Introduction

The coherent light sources in the extreme ultraviolet (XUV) spectral range allow the improvement of optical lithography, facilitate the optical visualization of nanoscale and subcellular systems, and allow monitoring of the movement of electrons in atoms, molecules, and nanostructured systems in real time. In addition, such radiation sources will contribute to creating a new analytical approach—resonance spectroscopy in XUV.

The most effective approach for generating coherent radiation in the XUV region is high-order harmonics generation (HHG) using ultrashort laser pulses. HHG from laser-matter interaction can be divided into four approaches using different mechanisms of laser-matter interaction: (a) the relativistic plasmas or totally ionized plasmas, (b) solid-state-structures and monoatomic crystals, (c) noble gases, and (d) laser plasmas. Among them, gases [1,2,3] and laser plasmas [4,5,6,7,8,9,10,11,12,13,14,15] are most frequently used options for HHG. In the latter case, the atomic targets are frequently used for the formation of laser-induced plasmas (LIP). Meanwhile, the molecular structures can also be used for HHG while properly ablated by laser radiation. This effect has been demonstrated in the case of complex molecular structures such as organic materials and food species [16], crystals [17], fullerenes [18], perovskites [19], as well as simple diatomic molecules [20].

Notice that carbon-containing plasma is the attractive medium for harmonics generation. Some compounds of carbon with other elements (carbides) can cause the combination of the advanced nonlinear optical properties of two components. The molecules containing metals and carbon (metal carbides) can be used for HHG and analyzed by different methods such as the application of chirped pulses, two-color pump, and different ablation methods. The interesting option here is the use of the nanoparticles (NPs) containing such molecules since NPs proved to enhance the harmonic yield.

The detailed analysis of chirped driving pulses application for HHG in argon gas jet are presented in [21] where the spectral and spatial changes in HHG are explained taking into account a differential contribution from the short and long quantum paths. In [22,23], the effects of ionization degree of argon in the semi-infinite gas cell by passing probing pulse were attributed to the self-phase modulation in the partially ionized media. This is particularly interesting, since we present the results of HHG in the partially ionized plasmas.

In this paper, for the first time to the best of our knowledge, we experimentally demonstrate the harmonic generation in the B_4_C and Cr_3_C_2_ NPs-containing plasmas and compare them with the SiC NP LIP. We examine various parameters of HHG in these LIPs. We also present the simplified two-color pump model calculations of HHG based on the strong field approximation.

## 2. Experimental Arrangements

**Figure 1:**
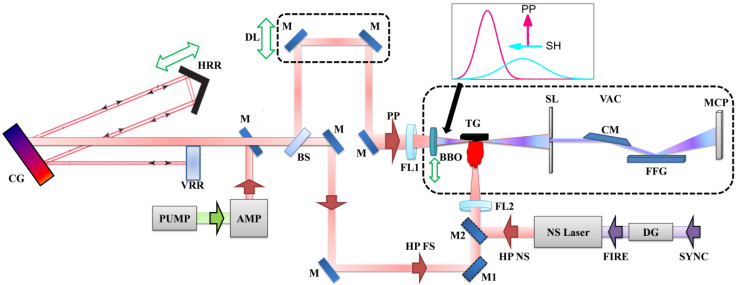
The experimental scheme of HHG. The uncompressed laser pulse after amplifier (AMP) is directed by a mirror (M) to the compressor comprising the compressing grating (CG), vertical retroreflector (VRR), and horizontal retroreflector (HRR). The compressed probing pulse (PP) propagates through the optical delay line (DL) and then focuses through a spherical focusing lens (FL1) inside the vacuum chamber (VAC) comprising the target chamber and XUV spectrometer. The focused pulses propagate through the 0.2 mm thick barium borate (BBO) crystal to generate a second harmonic (SH) beam for the two-color pump of plasma. Inset shows the relative positions of the SH (blue curve) and PP (red curve) and their polarizations after propagation of BBO. The femtosecond heating pulses (HP FS) are focused by a focusing lens (FL2) on the target (TG) to ignite the laser plasma. Optionally, nanosecond heating pulses (HP NS) are used. The delay between PP and HP NS is controlled by a digital delay generator (DG) triggered by a synchropulses (SYNC). The generated harmonics and fundamental radiation propagate through the slit (SL) and enter the XUV spectrometer comprising the cylindrical mirror (CM), flat-field grating (FFG), and micro-channel plate (MCP). M1 and M2 are the mirrors on flipping mounts for selecting either HP FS or HP NS.

Figure 1 shows the experimental scheme of HHG [24]. The femtosecond probing pulses (PP, 800 nm, 35 fs) were focused inside the LIP. The transmitted part of femtosecond beam after propagation of beamsplitter (BS) was used as one of the heating pulses (HP FS). The fluence of HP FS was tuned using a set of neutral density filters. The delay between HP FS and PP was controlled by variation of DL while being limited to 100 ns. This limitation was bypassed using another heating laser (NS laser; 1064 nm, 5 ns) to establish the electronically controlled delay. The generated harmonics were registered by XUV spectrometer.

Two metal and one non-metal carbide nanopowders [B_4_C (45–55 nm NPs), Cr_3_C_2_ (30–120 nm NPs), SiC (80 nm NPs), all Sigma-Aldrich] were prepared in the form of pressed tablets and inserted in the vacuum chamber for ablation.

## 3. Arrangement for Two-Color Pump of Plasma Using Chirp-Free and Chirped Pulses

We used both single-color pump (SCP, 800 nm) and orthogonally polarized two-color (OTC) pump (800 nm + 400 nm) of plasmas. In the latter case, the second wave was generated in the 0.2 mm thick barium borate (BBO) crystal (Figure 1). The second harmonic (SH) conversion efficiency was ~5% for the used PP intensities.

The chirps of PP, signs of chirps, and PP durations were modified using the variation of the distance between grating and horizontal retroreflector (HRR) inside the optical compressor (Figure 1). During experiments, the pulse duration was varied between 35 fs (for chirp-free conditions) and 70 fs and 130 fs (for chirped pulses). The pulse durations were measured by means of the autocorrelation technique. The pulse duration was determined in front of the entrance window of target chamber, so the last passing element was the 5 mm thick fused silica window. The low group velocity dispersion in this element did not notably increased the duration of 800 nm, 35 fs probing pulses.

**Figure 2:**
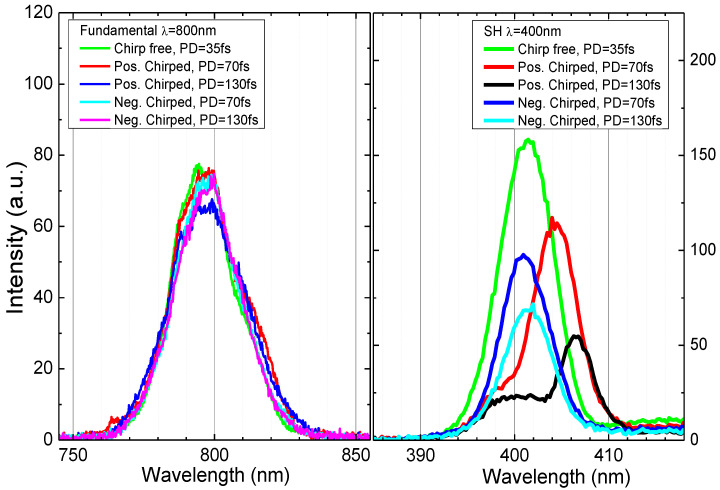
The fundamental ((**left**) panel) and second-harmonic ((**right**) panel) spectra of PP.

Figure 2 shows the spectra of 800 nm and 400 nm PP at variable chirps. As expected, despite the redistribution of the red and blue components, the spectrum of fundamental PP does not change (left panel of Figure 2). But in the case of the SH generated by chirp-free and chirped pulses, we observed the spectral shifts toward the corresponding chirp sign—relatively large redshift for positively chirped pulses and relatively small blue shift for negatively chirped pulses (right panel of Figure 2). The additional difference in the spectral shapes of 400 nm pulses can be explained by the changes in the optimal phase-matching conditions since the optimal orientation of BBO crystal was adjusted for the chirp-free pulse and then kept the same for the chirped pulses.

**Figure 3:**
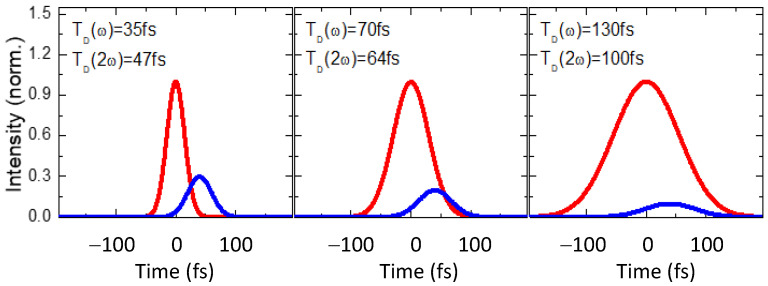
The modeled broadening of pulses and the relative time positions of SH pulses (blue curve) compared with the fundamental pulses (red curves) after passing through the 0.2 mm thick BBO crystal. The intensity of SH is increased with a factor of 4 for better visibility. T_D_(ω) and T_D_(2ω) are the pulse durations for fundamental and SH pulses.

Figure 3 shows three groups of temporal shapes of chirp-free and chirped 70- and 130-fs pulses determined using the relations from Ref. [25].

## 4. Results and Discussion

**Figure 4:**
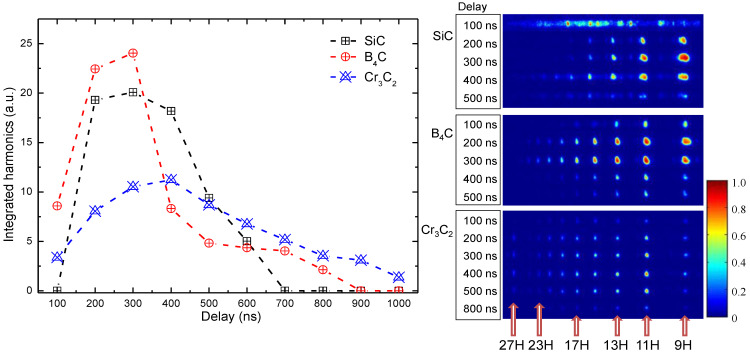
Integrated harmonics dependency on delay between HP NS and PP. Left panel presents dependency plot for integrated HHG signal for SiC NPs (black squares), B_4_C NPs (red circles), and Cr_3_C_2_ NPs (blue triangles). The right panel shows a few raw spectra of harmonics for the delays from 100 ns to 500 ns (SiC and B_4_C) and for Cr_3_C_2_ from 100 ns to 500 ns, but the last row presents HHG spectrum at 800 ns. Bottom numbers (9H–27H) correspond to the harmonic orders.

Since the used NP targets possess different molecular weights [w_mol_(SiC) = 40.09 g/mol, w_mol_(B_4_C) = 55.25 g/mol and w_mol_(Cr_3_C_2_) = 179.99 g/mol], we applied the same approach as in [26,27] by probing different LIPs at variable delays to observe the dependencies of harmonic yields on the molecular weight of elemental emitters. In the scheme with HP FS, where an optical delay line (Figure 1, DL) is used, its value was limited in our experiment by a maximum delay equal to D = 100 ns, which covers more than 30 m of actual distance on the optical table. Further increase of optical delay line was not practical. This limitation of maximum possible delay between HP and PP is bypassed by using a nanosecond laser synchronized through a digital delay generator (Figure 1, DG). HP NS fluence was equal to 2.6 J/cm^2^ for all three samples. The intensity of chirp-free PP with PD = 35 fs was maintained at *I_PP_* = 1.7 × 10^14^ W/cm^2^. The results of HHG at the delay range between 100 ns to 1 µs are shown in Figure 4.

A simplified consideration of the energy transition from heating pulse to the kinetic energy of LIP components assumes smaller velocity for heavier molecular components. This assumption was confirmed in [26,27] for the compounds constructed from carbon atoms (fullerenes, carbon nanotubes, diamond NPs, and others) and having much bigger weight distribution than metal carbide materials presented here. The left plot in Figure 4 shows an integrated harmonics signal. For lighter (SiC and B_4_C) NPs, the pronounced maximum is located in the 200–300 ns delay range, while for Cr_3_C_2_ NP the delay profile shape is more flattened, with the maximum located at 400 ns. The right panel in Figure 4 shows a set of raw images of harmonics at different delays. These images were collected from the phosphorous screen of MCP and captured at the same parameters of MCP and CCD, except for first row for SiC. This set of images is rescaled to be normalized to 1. It shows a suppression of HHG by emission of nanosecond plasma at early stages of LIP formation. At the given HP NS fluence, we observed this peculiarity only in the case of SiC. For this reason, the initial point at 100 ns for SiC curve is set to be equal to 0.

The raw images demonstrate evolution of harmonics spectra with spreading of LIP. Both cut-off positions and intensities of harmonics are changing. The cut-off positions were determined from the raw images obtained from the phosphorous screen of MCP. The last visible harmonic order was determined as a cut-off order. This procedure is considered as valid, since all raw images were captured at similar conditions (integration time, applied voltage to MCP and phosphorous screen, PP and HP repetition rate, and so on).

In the case of Cr_3_C_2_, the so-called resonance harmonic (RH) [28] appears starting at 200 ns delay. From distributions in Figure 4, we cannot conclude an unambiguous connection between the molecular weight of ablated material and maximum for HHG yield. One of the reasons could be a difference in LIP formation from the bulk sample and the pressed nanoparticles made of the same material. Another uncertainty is a “comparability” of HHG spectra obtained from the LIPs produced by heating pulses of different (nanosecond and femtosecond) pulse duration. Under “uncertainty in “comparability” we mean a non-negligible difference in HHG spectra, cut-off position, and harmonics distribution obtained from the LIP produced by nano- or femtosecond heating pulses. In each case (i.e., for ns and fs HPs) we carried out the optimization of HHG yield by determining the proper fluencies of HP on the targets surface, which will be substantially different for each timescale of heating pulses. The problem of “comparability” is also related with the delay between HP and PP since for producing large delays we used two synchronized lasers, while for shorter delays it was convenient to use the optical delay line.

Recently, we demonstrated HHG from complex molecular LIP’s produced by femto-, pico-, and nanosecond HP [24]. It was shown that by selection of proper HP fluencies and delays between HP and PP, the general HHG features such as cut-off and harmonic distribution can be reproduced for the PPs with the same intensity. The same is valid here. In the following part of this paper, we present the HHG spectra obtained using femtosecond HP with optimal fluence equal to 0.28 J/cm^2^ and fixed delay (70 ns) produced by DL (Figure 1). Previously, the optimal delay for HP FS was found much smaller than for HP NS (70 ns vs. 200–400 ns) [24]. This effect was also observed in the present study. Similar observation was reported in [29].

**Figure 5:**
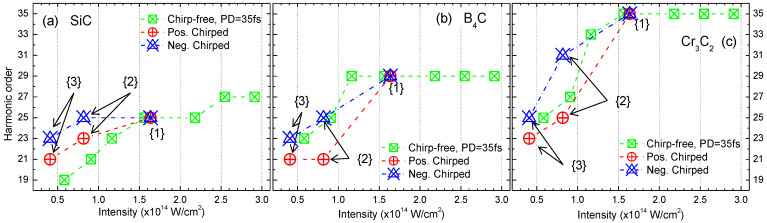
Cutoff position dependencies for (**a**) SiC, (**b**) B_4_C, and (**c**) Cr_3_C_2_ NP plasmas at different *I_PP_* intensities for chirp-free 35 fs pulses (crossed green squares), positively chirped pulses (crossed red circles), and negatively chirped pulses (crossed blue triangles). Point {2} and {3} designate chirped pulses with the same pulse durations, 70 fs and 130 fs correspondingly, but possessing opposite signs of chirp. Point {1} designates share points where red and blue curves achieve chirp-free 35 fs pulse by tuning distance between CG and HRR (see Figure 1).

In our experiment, the change from chirp-free 35 fs pulses to the positively/negatively chirped pulses was performed by tuning a distance between CG and HRR (see Figure 1) symmetrically, from the point of view of the pulse duration measurements using autocorrelation technique. Notice that pulse energy was not changed during the variations of laser chirp. So, we have the pairs of pulses with the same 70 fs and 130 fs pulse durations but possessing opposite signs of the chirp. Figure 5 shows the comparison of cutoff harmonic orders in three scenarios. First scenario, plotted with green crossed squares is the variation of cutoff position with the change of chirp-free 35 fs pulse energy. The pulse’s energy was tuned by the controlled pump of laser amplifier (PUMP and AMP in Figure 1). Starting from the maximal intensity defined for each material, one can see the saturation effect, when increasing the *I_PP_* does not increase the cutoff order. Other two scenarios plotted with red and blue crossed symbols in Figure 5 present the dependencies of cutoff position in the case of variations of chirp. Red symbols correspond to the path of increasing positive chirp by points {1}→{3} and blue—the path of increasing negative chirp by points {1}→{3}. Point {2} and {3} designate chirped pulses with the same measured by autocorrelation technique pulse durations, 70 fs and 130 fs correspondingly, but opposite signs of chirp. Point {1} designates share points where red and blue curves achieve chirp-free 35 fs pulse by tuning a distance between CG and HRR. For all three materials, the initial point {1} is chosen *I_PP_* = 1.65 × 10^14^ W/cm^2^. The pulse energy along paths {1}→{3} was not changed.

In the case of B_4_C and Cr_3_C_2_ NPs (Figure 5b,c), starting intensity point {1} is placed close to or into saturation range and we can see the common behavior for chirp-free (green crossed squares) and negatively chirped paths, while moving along the positive sign of chirp the path reduced the cutoff position. In the case of SiC NPs (Figure 5a), the starting point {1} is placed below the saturation intensity. While the negative/positive chirp paths demonstrate similar pattern, the relative position for chirp-free path was changed.

Concerning different cut-off scaling in the case of the positive and negative chirps, it could be due to the uncertainty in definition of the peak intensity for chirped pulses. The duration of those pulses obtained from autocorrelation measurements was then applied for the calculation of intensities while assuming the Gaussian shape of the chirped pulses, which is strictly speaking not completely valid.

Special comments can be made for the RH of Cr_3_C_2_ (29H). For chirp-free path (green crossed squares, Figure 5c), this harmonic was always presented down to the lowest *I_PP_* intensities, while observed cut-off of HHG plateau is moving to the lower harmonics (from 35th to 23th order). We do not consider 29H as real cutoff position in these scenarios, since the appearance of resonance harmonics cannot be described by the three-step relation (Equation (3)). For negative/positive chirp paths, RH is presented only for negative chirp scenario, as will be demonstrated below.

Together with cutoff scaling, the chirped PPs led to observable spectral effects [30,31,32]. Combined with OTC, the application of chirped PPs sufficiently enriched the observed HHG spectra.

Below we present the experimental results of HHG with chirped PPs using SCP and OTC schemes together with the simplified two-color pump model calculations based on the strong field approximation, commonly referred to as the Lewenstein HHG model [33]. HHG spectra are calculated from Fourier transform of the time-dependent induced dipole moment components:

(1) $${\left|D_{k}\left(\omega \right)\right|}^{2}={\left|\frac{1}{2\pi }\int dte^{-i\omega t}d_{k}\left(t\right)\right|}^{2}$$

where time-dependent dipole moment ***d***(*t*) is:

(2) $$d\left(t\right)=i\int_{0}^{\infty }d\tau {\left(\frac{\pi }{\varepsilon +i\tau /2}\right)}^{\frac{3}{2}}r^{*}\left(t\right)\left[\overset{z}{\underset{k=x}{\sum }}r_{k}\left(t-\tau \right)\cdot E_{k}\left(t-\tau \right)\right]e^{-iS\left(t,\tau \right)}+c.c.$$

Here *E_k_*(*t*) is the *k*-th component of electric field strength vector. The dipole matrix elements ***r***(*t*) are taken for hydrogen-like wave function for simplicity:

(3) $$r\left(t\right)=\langle p_{st}-A\left(t\right)|r|0\rangle =-i\frac{4\alpha^{5/2}}{\pi^{5/2}}\frac{p_{st}-A\left(t\right)}{{\left({\left(p_{st}-A\left(t\right)\right)}^{2}+\alpha^{2}\right)}^{3}}$$

(4) $$S\left(t,\tau \right)=\frac{1}{2}\overset{t}{\underset{t-\tau }{\int }}{\left(p_{st}-A\left(t^{\prime }\right)\right)}^{2}dt^{\prime }+I_{p}\tau$$

The parameter *α* in Equation (3) is taken as *α*^2^ = 2*I_p_*. The ionization potentials *I_p_* for each LIP were deduced empirically from Figure 5 for the point {1}. *S*(*t*,*τ*) is the quasiclassical action taken in the stationary point ***p****_st_* defined by equation:

(5) $$p_{st}\left(t,\tau \right)=\frac{1}{\tau }\overset{t}{\underset{t-\tau }{\int }}A\left(t^{\prime }\right)dt^{\prime }$$

Here ***A***(*t*) is the vector potential of applied field. In the model calculations, the vector of electrical field strength ***E***(*t*) is defined for two-color pump as follows:

(6) $$E\left(t\right)=E_{\omega }\cos\left(\omega t\right)+E_{2\omega }\cos\left(2\omega t+\varphi_{0}\right)$$

Here vectors ***E****_ω_* and ***E***_2*ω*_ define the mutual orientation of ω and 2ω pumping fields and *φ*_0_ is a phase shift between two fields. We numerically considered the parallel and orthogonal orientations of vectors, which correspond to the parallel two-color (PTC) pump and OTC pump.

**Figure 6:**
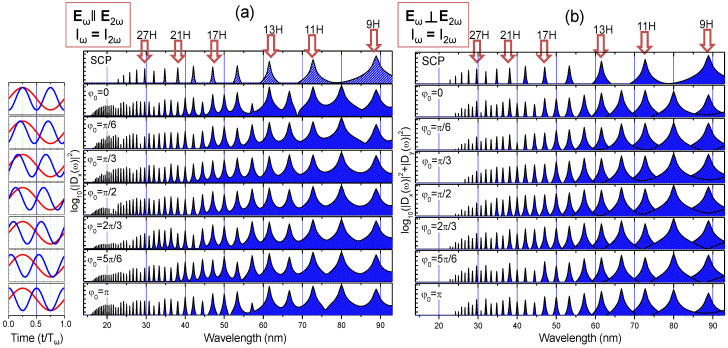
Calculated HHG spectra for PTC (**a**) and OTC (**b**). The *φ*_0_ designates a relative phase shift for ω-2ω fields, schematically presented in first column by red (800 nm) and blue solid (400 nm) lines. The intensities of ω and 2ω are equal, while the orientation of polarization vectors is parallel in panel (**a**) and orthogonal for panel (**b**). Upper panels (SCP) present the calculated HHG with only 800 nm field.

**Figure 7:**
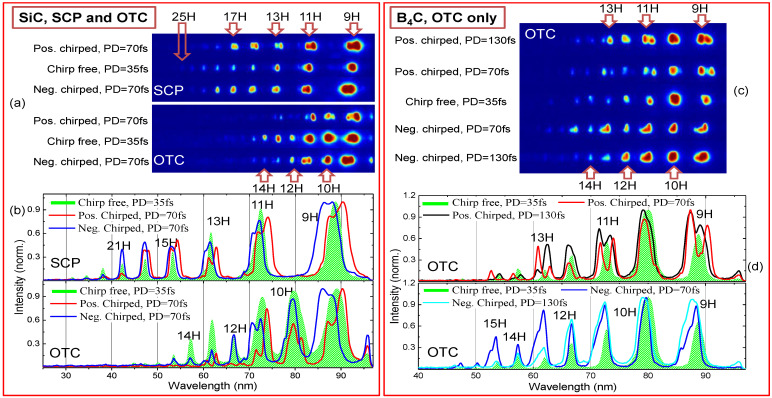
HHG for SiC and B_4_C NP LIPs with chirped and chirp-free SCP and OTC. Panel (**a**,**c**) show raw MCP images for SCP and OTC using 0.2 mm thick BBO crystal. Panel (**c**) presents only OTC for B_4_C LIP. Panels (**b**,**d**) present profiles plots for corresponding images.

Figure 6 shows the results of HHG calculation where the values of *I_p_* and *I_ω_* correspond to the experimental results of SiC LIP presented in Figure 7. The ionization potential *I_p_* was deduced for *I_ω_* = 1.65 × 10^14^ W/cm^2^ and the cut-off position from Figure 5a corresponded to *I_p_* = 9 eV. Figure 6 presents the calculations using Equation (1) in the case of equal intensities of ω and 2ω fields (*I_ω_* = *I*_2*ω*_) while their polarizations were orthogonal or parallel to each other. For simplicity, ***E****_ω_* is always oriented along the *x*-axis, while ***E***_2*ω*_ is oriented along either *x*-axis or *y*-axis. For comparison, the 800 nm pump is presented with top row HHG spectra (Figure 6, SCP). Harmonic spectra with different phase shifts *φ*_0_ are presented between 0 and π with a π/6 step. The corresponding profiles of ω and 2ω fields are shown in the first column of Figure 6 with red and blue solid lines in a time range of single period for fundamental field.

In the case of PTC (Figure 6a), only *D_x_* component from Equation (4) is nonzero, while for OTC (Figure 6b) two components persist, *D_x_* and *D_y_*. Correspondingly, the resulted HHG spectra are presented as the logarithms of sum |*D_x_*|^2^ + *|D_y_*|^2^. Simultaneous presence of the second field leads to appearance of all even and odd orders of harmonics for both orientations. Calculations show the importance of orientation of ***E****_ω_* and ***E***_2*ω*_ vectors. For PTC, one can see the enhancement of cutoff position from 27th order to 41th–43th orders, while, for OTC, the cutoff position remains unchanged with some intensity modulations at the end of the plateau. Changing of *φ*_0_ leads to the modulation of harmonic intensities at the cutoff region of plateau (in the case of *φ*_0_ = π/2 to *φ*_0_ = 5π/6 for PTC, Figure 6a). In real experiments, this could be observed as the variation of cutoff. A similar minimum is observed for OTC (Figure 6b, *φ*_0_ = π/3).

Figure 7 presents the harmonic spectra during probing of SiC and B_4_C NP LIPs using chirp-free and chirped pulses. Green-filled profiles in panels (b) and (d) correspond to the chirp-free pulses with a pulse duration of 35 fs and *I_ω_* = 1.65 × 10^14^ W/cm^2^ in SCP and OTC configurations. We estimated the intensity of SH pulses to be *I*_2*ω*_~9 × 10^12^ W cm^−2^. The most observable peculiarity is a strong reduction of cutoff position for the OTC pump. In the case of SiC NP LIP, it moved from 25H to 15–16H and for B_4_C NP LIP it moved from 29H to 17H, despite relatively small SH intensity.

**Figure 8:**
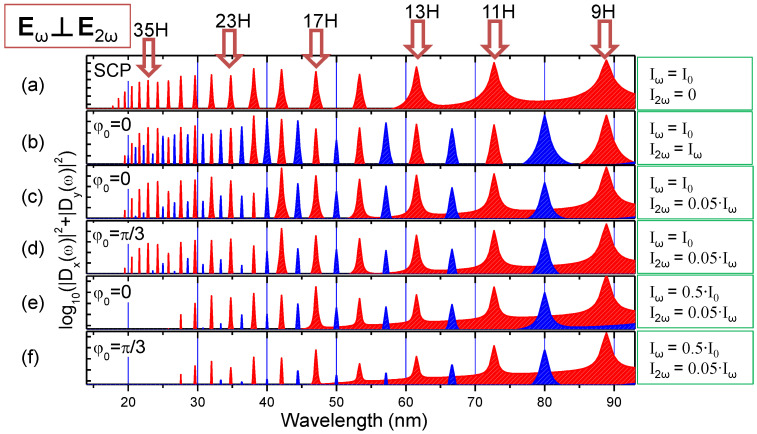
Calculated HHG spectra for PTC with *I_p_* = 21.5 eV were empirically determined through Equation (3) for Cr_3_C_2_ LIP. Panel (**a**) shows the calculated spectrum of harmonics in the case of single-color pump. Through panels (**b**–**f**), different relations between *I_ω_*, *I*_2*ω*_, and *φ*_0_ were considered. Here *I*_0_ = 1.65 × 10^14^ W/cm^2^. SCP row presents the calculated HHG with only 800 nm field.

The model calculations (Figure 6b) for equal ω and 2ω fields do not predict such behavior. Intensity ratios for model calculations were determined using the pulse energy and duration measurements for fundamental 800 nm and SH using the estimations described in Ref. [25]. We did not exactly measure the phase difference, but instead modeled the variations of HHG spectra using simplified model in the case of chirp-free pulses. In experiment, the reduction of cut-off was observed in the case of the chirp-free pulses. This process was not predicted even for equal ω and 2ω intensities when the strongest mutual influence of two waves is expected. Some reduction of cut-off is predicted for the phase difference around π/3, so this phase difference was used for simulating Figure 8 in the case when ω and 2ω intensities were chosen close to the experimental one.

**Figure 9:**
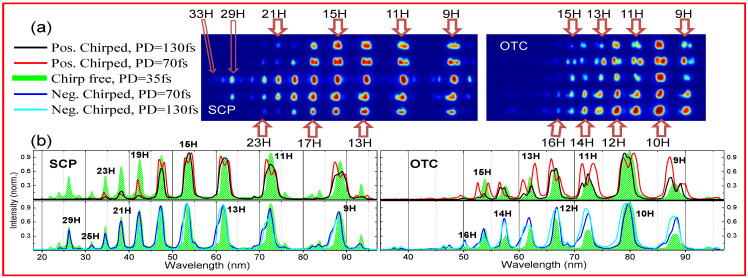
HHG for Cr_3_C_2_ NP LIP with chirped and chirp-free SCP and OTC. Panel (**a**) shows raw MCP images for SCP and OTC using 0.2 mm BBO crystal. Panels (**b**) present profile plots for corresponding images.

The corresponding calculations for the scenarios with a small 2ω field are presented in Figure 8c,d and do not demonstrate strong reduction of cutoff order in the case of chirp-free PPs. The comparison of Figure 8b with Figure 8c,d confirms that the presence of even a small portion of SH in the case of OTC is sufficient for the appearance of the even orders of harmonics. However, the theory predicts weak influence of the fundamental wavelength on the cutoff position, which is defined by HHG plateau. The calculations for Figure 8 are taken for “empirical” Cr_3_C_2_ LIP, which allowed defining *I_p_* = 21.5 eV. The corresponding experimental results for Cr_3_C_2_ NP LIP are shown in Figure 9.

Even stronger deviation in the case of OTC scheme was observed in the case of combination with positively/negatively chirped PPs. In Figure 7c,d, the black/red and blue/cyan solid line profiles present the results of interaction of 130/70 fs chirped pulses with B_4_C NP LIP.

In Figure 8e,f we numerically emulated a two-fold drop of intensity for 70 fs PPs, which demonstrates a suppression of even-order harmonics, especially in the vicinity of the cutoff region. In contrast, Figure 9a (OTC panel) shows the inverse distribution of harmonics, where even-order harmonics become stronger or equal to odd orders.

We also observed the either red or blue spectral shifts, as well as the appearance of harmonics splitting into two redder/bluer components. This was strongly expressed for the intermediate chirped pulses with 70 fs duration (see Figure 7a,c and Figure 9a) and tended to appear for positively chirped PPs. In the case of Cr_3_C_2_ NP LIP for OTC with 70 fs positively chirped pulse (see Figure 9a), we observed splitting of odd and even orders of harmonics. Note that the simplified model presented by Equation (2) does not consider the chirp effect and hence cannot be used for the reproduction of observed spectral shifts. Moreover, the material itself was modeled using the condition when the parameter *α* in Equation (3) was taken as *α*^2^ = 2*I_p_* where *I*_p_ was deduced empirically from Figure 5 for the cut-offs at non saturated chirp-free pulse intensities.

Splitting and tuning of harmonics could not be explained solely by simple assumption of the main role of the leading front of laser pulse as a source responsible for the variation of the spectral position of harmonics, since the spectral shift in that case should be proportional to the harmonic order. Instead, it was demonstrated in [21,23] for short and long quantum orbits the appearance of non-equal spectral shift, since long orbits experienced sufficiently stronger shift with PP’s chirp. In [23], the converting gas medium was partially ionized with strong PP (i.e., at the intensities above 10^15^ W/cm^2^), resulting in the self-phase modulation. We used sufficiently weaker PP (below 2 × 10^14^ W/cm^2^), while the medium (plasma) was already partially ionized during ablation. We clearly observed the negative/positive spectral shift of the one of the splitted component in the case of SiC LIP (Figure 7a,b; see the profiles for positive/negative chirped 70 fs PPs) and B_4_C LIP (Figure 7c,d; see the profiles for positively chirped PPs). These observations could be attributed to differential contributions from short and long trajectories. In the case of Cr_3_C_2_ NP LIP, the spectral shift/splitting is expressed as stronger for positive chirped 70/130 fs PPs (Figure 9, black/red profiles). Among the three materials, Cr_3_C_2_ LIP has highest cutoff and hence highest effective ionization potential in Equation (3), so one can assume smaller degree of ionization and accompanied spectral shift for the negatively chirped PPs.

Another spectral peculiarity is related with the presence of 29th RH (λ = 27.6 nm) of 800 nm pump in the case of Cr_3_C_2_ NP LIP. It demonstrates the chirp-assisted spectral tuning since RH was presented only in the case of the chirp-free 35 fs and negatively chirped 70 fs pulses (see green and blue profiles in Figure 9b), while with an increasing amount of chirp for 130 fs negatively chirped pulse (cyan profile), 29th harmonic disappeared. For OTC pump, 29th harmonic was not present for all combinations of chirp/pulse durations. This fact, in connection with model calculations and variation of intensity for chirp-free SCP (Figure 5c), allows us to assume that in the presented OTC scheme, placing BBO crystal on the beam path after focusing lens leads to strong distortion of 800 nm wavefront. Correspondingly, the intensity in the focal spot drops, thus reducing the cutoff position.

## 5. Conclusions

We reported the study of carbide-containing SiC, B_4_C, and Cr_3_C_2_ nanoparticles as media for high-order harmonics generation during single-color pump and orthogonal polarized two-color pump with chirped and chirp-free pulses. Simplified two-color strong field approximation model of HHG in the case of PTC and OTC was used for comparison with experimental results. Calculations demonstrated the role of the polarization orientations of ω and 2ω fields. It was shown that even a small 2ω field leads to appearance of all odd and even harmonics. The changing of maximum cutoff energy is predicted only for PTC configuration, while in the case of OTC the one is shown to be defined only by the fundamental ω field. The observed contradiction allows us to assume that, in the presented OTC scheme, placing BBO crystal on the beam path after the focusing lens leads to strong distortion of the fundamental beam wavefront and the corresponding drop of the intensity in the focal spot area, thus leading to the reduction of the cutoff position.

## Data Availability

The data that support the findings of this study are available on request from the corresponding author.
